# An experimental model for studying the biomechanics of embryonic tendon: Evidence that the development of mechanical properties depends on the actinomyosin machinery

**DOI:** 10.1016/j.matbio.2010.08.009

**Published:** 2010-10

**Authors:** Nicholas S. Kalson, David F. Holmes, Zoher Kapacee, Iker Otermin, Yinhui Lu, Roland A. Ennos, Elizabeth G. Canty-Laird, Karl E. Kadler

**Affiliations:** a Wellcome Trust Centre for Cell-Matrix Research, Faculty of Life Sciences, University of Manchester, Michael Smith Building, Oxford Road, Manchester M13 9PT, UK; b Faculty of Life Sciences, University of Manchester, Stopford Building, Oxford Road, Manchester M13 9PT, UK

**Keywords:** CVF, cell volume fraction, the fraction of the construct occupied by cells, ECM, extracellular matrix, ECMT, embryonic chick metatarsal tendon, EM, electron microscopy, FACS, fluorescence activated cell sorting, FVF, fibril volume fraction, the fraction of the construct occupied by collagen fibrils, NMMII, non-muscle myosin II, PBS, phosphate buffered saline, Bio-artificial tendon, Collagen, Elasticity, Electron microscopy, Extracellular matrix, Myosin, Fibril, Fibrin, Tension

## Abstract

Tendons attach muscles to bone and thereby transmit tensile forces during joint movement. However, a detailed understanding of the mechanisms that establish the mechanical properties of tendon has remained elusive because of the practical difficulties of studying tissue mechanics *in vivo*. Here we have performed a study of tendon-like constructs made by culturing embryonic tendon cells in fixed-length fibrin gels. The constructs display mechanical properties (toe–linear–fail stress–strain curve, stiffness, ultimate tensile strength, and failure strain) as well as collagen fibril volume fraction and extracellular matrix (ECM)/cell ratio that are statistically similar to those of embryonic chick metatarsal tendons. The development of mechanical properties during time in culture was abolished when the constructs were treated separately with Triton X-100 (to solubilise membranes), cytochalasin (to disassemble the actin cytoskeleton) and blebbistatin (a small molecule inhibitor of non-muscle myosin II). Importantly, these treatments had no effect on the mechanical properties of the constructs that existed prior to treatment. Live-cell imaging and ^14^C-proline metabolic labeling showed that blebbistatin inhibited the contraction of the constructs without affecting cell viability, procollagen synthesis, or conversion of procollagen to collagen. In conclusion, the mechanical properties *per se* of the tendon constructs are attributable to the ECM generated by the cells but the *improvement* of mechanical properties during time in culture was dependent on non-muscle myosin II-derived forces.

## 1 Introduction

The ability of tendons to transmit tensile forces is acquired during embryonic development in preparation for ambulatory movement at birth, especially in birds, reptiles and some mammals. However, the mechanisms that establish the mechanical properties of tendon during embryogenesis have been difficult to determine because of impracticalities of studying cell biomechanics *in vivo*. In a previous study we showed that day 13 embryonic chick metatarsal tendon (ECMT) cells synthesize a tendon-like construct when cultured in fixed-length fibrin gels (Kapacee et al., 2008). A motivation for our present study was to evaluate the mechanical properties of the constructs, and then, if appropriate, to begin to ask questions about the contributions of cells and ECM to the mechanical properties of the constructs. In particular, we wanted to know if the force-generating machinery of the cell (the actinomyosin system) is important in establishing and/or maintaining the mechanical properties of the tissue.

Tendons comprise a dense extracellular matrix (ECM) of predominately collagen fibrils arranged in parallel bundles along the tissue. The fibrils are the primary tensile element in vertebrate tissues, are indeterminate in length, range in diameter from ~ 12 to ~ 500 nm (depending on tissue and stage of development) (Parry et al., 1978), and can fuse (tip to shaft) to generate Y-shaped branched fibrils in tendon (Starborg et al., 2009). During tendinogenesis the collagen fibrils are deposited into cell-surface channels that are contiguous from one cell to another (Birk and Trelstad, 1985, 1986; Richardson et al., 2007). The cells exhibit fibripositors, which are actin-rich plasma membrane protrusions that contain the narrow collagen fibrils (Canty et al., 2004; Canty et al., 2006). Furthermore, as tendon development proceeds, the volume fraction of collagen increases until in postnatal tissue the collagen fibrils are interspersed with relatively few tendon cells (tenocytes) (McBride et al., 1985, 1988).

However, the ability of the cells to synthesize collagen fibrils does not fully account for the tensile and viscoelastic properties of tendon. Chemical evidence suggests that trivalent intermolecular crosslinks stabilize the fibrillar structure of collagen and thereby stiffen the tendon (Bank et al., 1999; Ng et al., 1996). Other studies have shown that stiffness and Young's modulus increase during tendon ageing (O'Brien et al., 2010), but the molecular origin for the development of stiffness is unclear. Studies of genetically defective mice have suggested that decorin (a small leucine rich proteoglycan that locates to the surfaces of collagen fibrils) contributes to tendon viscoelasticity (Robinson et al., 2004) whilst another study suggests that the glycosaminoglycan side chains of small leucine rich proteoglycans (such as decorin) do not mediate either dynamic elastic behaviour or viscoelastic properties of tendon (Fessel and Snedeker, 2009). Still further studies have demonstrated integrins interacting with collagen (for a review of integrin function see Askari et al. (2009) and Humphries et al. (2006)) but their contribution to the mechanical properties of tendon is unclear. It seems likely, therefore, that several cellular and ECM factors work in unison to establish the tensile and viscoelastic properties of tissues, such as tendon. However, identifying the different contributory factors is complicated by the difficulty of perturbing the activities of molecules *in vivo* and because *ex vivo* tissues degenerate (Egerbacher et al., 2008) as cells release catabolic enzymes and degrade the ECM (Lavagnino and Arnoczky, 2005).

The tendon constructs used in this study were prepared as previously described using fibrin as the provisional matrix (Kapacee et al., 2008). Fibrin has been used to culture a variety of cells including muscle cells (Huang et al., 2005, 2006, 2007) and fibroblasts (Harris et al., 1984; Steinberg et al., 1980; Tuan and Grinnell, 1989). Collagen has also been used with success to study the migration and force-generating abilities of fibroblasts (see Grinnell and Petroll (2010), Marquez et al. (2006), Wakatsuki and Elson (2003), Wille et al. (2006) and references therein). Whilst much is known about the mechanical properties of contractile fibroblasts, it remains controversial how pulling forces exerted by cells contribute to the assembly of a collagen matrix and establish the mechanical properties of the matrix. We chose not to use collagen as the provisional matrix because the presence of pre-existing fibrils would complicate interpretation of our results on the ultrastructure of the collagen fibrils synthesized by the cells. The fact that the provisional matrix was fibrin (lacking any pre-existing collagen) meant that the ultrastructural and biomechanical properties were the direct result of the *de novo* matrix assembled by the cells and not of matrix resulting from remodeling of pre-existing collagen. We used the tendon constructs to test the hypothesis that the actinomyosin machinery has a role in establishing the mechanical properties of newly-synthesized collagen fibril matrix. Non-muscle myosin II (NMMII) is an adenosine triphosphate-driven molecular motor, which through the interaction with the actin cytoskeleton, forms part of the force-generating machinery for most non-muscle cells (Jay et al., 1995). Blebbistatin is a small molecule inhibitor (Limouze et al., 2004) that shows high affinity and selectivity toward NMMII adenosine triphosphatase activity with minimal effects on other types of myosin (Allingham et al., 2005). The results showed that inhibition of NMMII by blebbistatin stops the *development* of mechanical properties but does not change the *existing* mechanical properties of the tendon constructs.

## 2 Results

### 2.1 Reproducible production of tendon constructs

Three hundred tendon constructs were made from 13-day ECMT cells of which 285 formed successfully. Fifteen constructs formed a loose gel between the pins and were discarded. Tendon constructs (10 mm in length) maintained a mean diameter of 0.93 ± 0.02 mm corresponding to a mean transverse area of 0.68 ± 0.03 mm^2^ over the first 7 days in culture.

### 2.2 Tendon constructs develop near-identical mechanical properties to embryonic chick metatarsal tendon

**Fig. 1:**
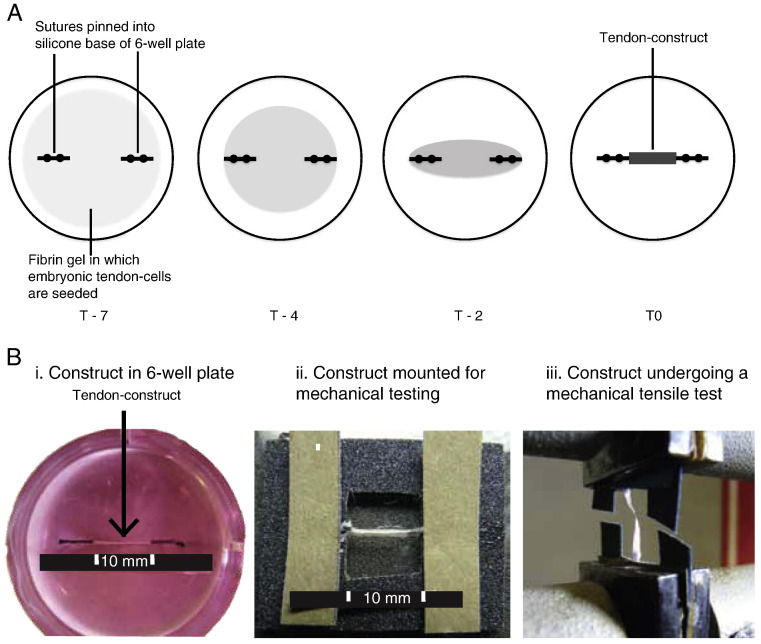
Tendon construct formation and mechanical testing. (A) Schematic representation of the formation of a tendon construct. From left to right, during 7 days in culture, cells contract the fibrin gel, replace the fibrin with collagen fibrils, and form a mechanically robust linear construct. (B) Left to right, images of a T0 construct in the culture dish, mounted on a sand paper window frame, and being mechanically tested to failure.

**Fig. 2:**
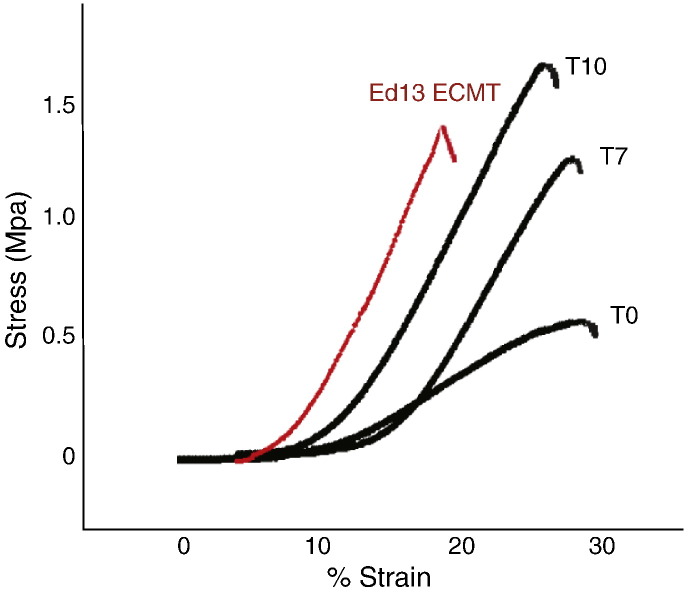
Typical stress–strain curves for T0, T7 and T10 tendon constructs shown in comparison with data from 13-day ECMT. All curves show the distinct regions (toe, linear and failure) characteristic of tendon. The tendon constructs show a progressive increase in both stiffness (gradient of linear region) and breaking stress with time from T0 to T10.

Tendon constructs failed in their mid-substance during tensile testing (Fig. 1). Stress–strain curves exhibited the 3 regions characteristic of ECMT, i.e. toe–heel, linear and failure regions (Fig. 2). The toe–heel region is thought to originate from straightening of crimped collagen fibrils, which were previously shown by plane polarized light microscopy to occur in the tendon constructs (Kapacee et al., 2008). The curves enabled calculation of mechanical parameters including ultimate tensile stress (UTS), elastic modulus and failure strain. For comparison, the UTS, elastic modulus and failure strain of 13-day ECMT were measured.

**Fig. 3:**
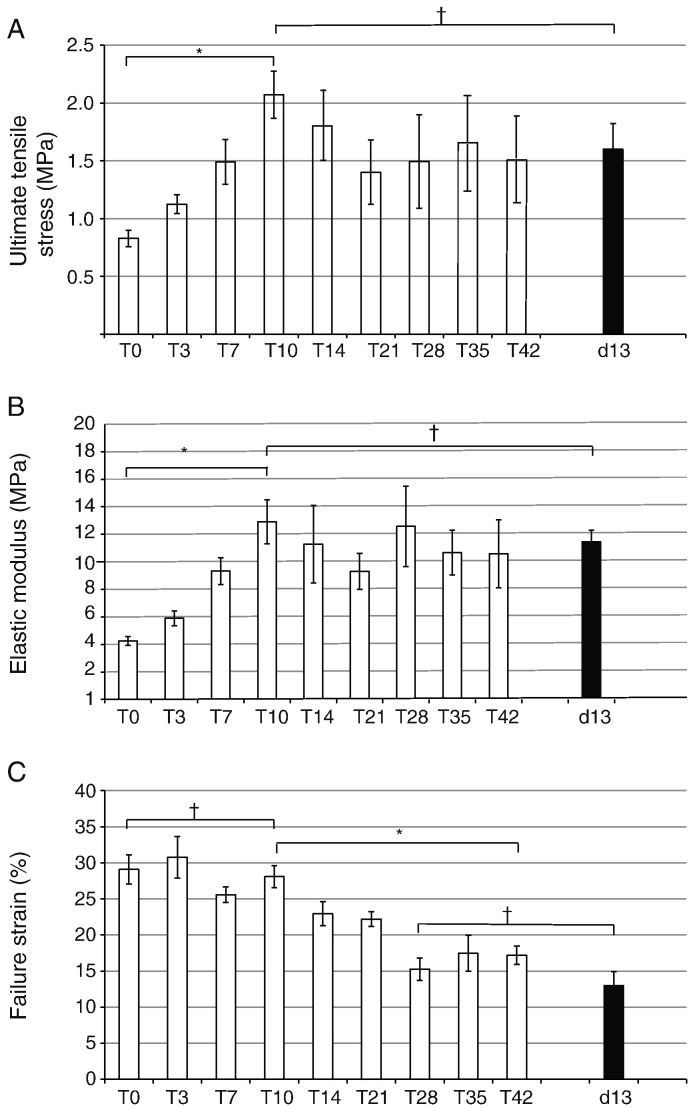
Summary of data for the mechanical properties of tendon constructs (from T0 to T42) shown compared with the corresponding data on 13-day ECMT. (A) The ultimate tensile stress (UTS) of tendon constructs increased over the initial culture period from 0.87 ± 0.07 MPa at T0 to 2.10 ± 0.20 at T10 (*p* < 0.05). After T10 the UTS stabilized, reaching 1.52 ± 0.21 at T42 (*p* < 0.05). The UTS in the T10 construct was similar to that exhibited by 13-day ECMT, which had a value of 1.59 ± 0.19 MPa. (B) The elastic modulus of tendon constructs also increased in the initial culture period from 4.25 ± 0.32 MPa at T0 to 12.91 ± 1.60 MPa at T10 (*p* < 0.05). The elastic modulus stabilized after T10, reaching 10.82 ± 2.47 at T21 (*p* < 0.05). The elastic modulus at T10 was similar to that exhibited by 13-day ECMT, which had a value of 11.47 ± 0.82. (C) Failure strain was greater for the tendon constructs than for ECMT up to T21, after which the constructs showed a similar failure strain to that exhibited by 13-day ECMT. ^⁎^*p* < 0.05, ^†^*p* > 0.2.

*Ultimate tensile stress (UTS)*: Measured as the stress at which the constructs failed, this was a direct measure of the strength of the constructs. As shown in Fig. 3A, the constructs increased in UTS during T0 to T10 (10 days after the formation of a construct). During the following few days the UTS decreased to ~ 70% of the maximum value (at T10) and remained constant until at least T42 (the longest time point studied). Importantly, the UTS of older tendon constructs was not significantly different from that of 13-day ECMT.
*Elastic modulus*: Measured as the change in stress per unit strain over the linear region of the stress–strain curve, the elastic modulus was a measure of the elastic deformability of the constructs. As shown in Fig. 3B, the constructs increased in elastic modulus from T0 to T10, and remained approximately constant until at least T42. Importantly, the elastic modulus of older tendon constructs was not significantly different from that of 13-day ECMT.
*Failure strain*: Measured as the increase in length of the construct as a percentage of original length at the point of failure, this gave an indication of the displacement (e.g. sliding) of structures (e.g. collagen fibrils) within the construct because of deformation. The results showed that T0 constructs exhibited ~ 2-fold greater failure strain than 13-day EMCT, suggesting that in younger constructs there is considerable sliding of tensile components prior to failure. However, the constructs consolidated with time in culture (e.g. during T0 to T42) and the failure strain decreased to a value that was similar to that of 13-day EMCT (Fig. 3C).

### 2.3 Tendon constructs develop a collagen fibril-based matrix similar to chick embryonic tendon

**Fig. 4:**
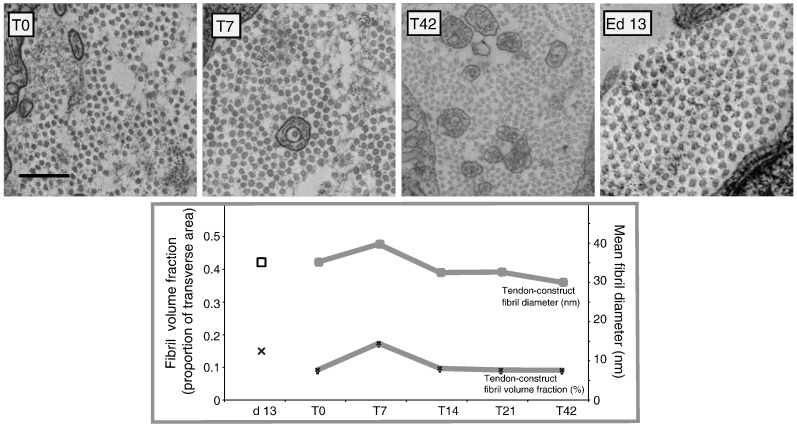
Transmission electron microscopy of tendon constructs and embryonic chick metatarsal tendon (ECMT). Typical images of transverse sections of tendon constructs at T0 (A), T7 (B) and T42 (C) and 13-day ECMT are shown. Plots of mean fibril diameter and mean fibril volume fraction as a function of time in tendon constructs and 13-day ECMT are shown.

Next, we wanted to know if the changes in mechanical properties of the constructs during time in culture were related to changes in the diameter or packing of the collagen fibrils. Therefore we examined transmission electron micrographs of transverse sections of tendon constructs at time points matched to the biomechanical tests. The results are summarized in Fig. 4. The mean collagen fibril diameter initially increased from 33.3 ± 0.5 nm to 38.7 ± 0.1 nm (*p* < 0.05, n = 1000 per sample) during T0 to T7 and then decreased to 27.1 ± 0.1 nm at T42. Likewise, fibril volume fraction (FVF) increased approximately two-fold from 0.09 ± 0.01 at T0 to 0.17 ± 0.01 at T10 but then decreased to 0.09 ± 0.008 at T42. The FVF values at T0 and T42 were not significantly different from each other. Fibril diameter and FVF closely matched that of 13-day ECMT (33.5 ± 0.01 nm and 0.13 ± 0.01 respectively, Fig. 4). The results showed that the changes in mechanical properties of the constructs during time in culture were not fully explained by changes in collagen fibril diameter or FVF.

### 2.4 Tendon-construct cell number increases to a maximum 2 days prior to maximum contraction and then decreases to a steady value at T21

**Fig. 5:**
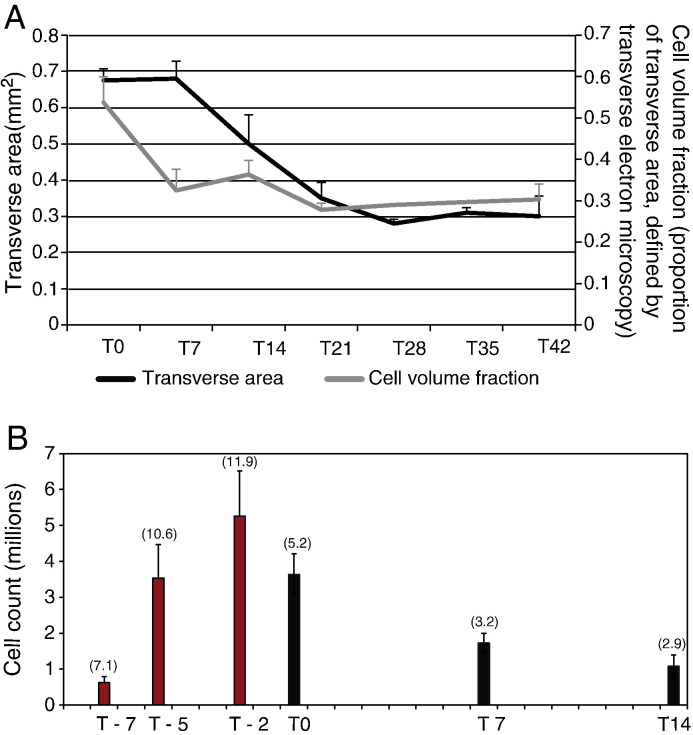
Comparison of construct transverse area, cell volume fraction and cell number. (A) Plots of transverse area and cell volume fraction for the tendon construct as a function of days in culture. The constructs initially maintain a constant transverse area from T0 to T7. Prolonged culture results in a 43% decrease in transverse area over the next 14 days. In contrast, the cell volume fraction showed an initial 39% decrease from T0 to T7 before stabilizing at a value of ~ 0.3 by T21. (B) Quantification of the live-cell population of tendon constructs over time in culture. Bracketed numbers at the head of the bars are the percentage of cells in the S-phase. Red bars correspond to pre-contraction of the tendon construct, black bars to post-contraction.

To determine the effects of cell volume fraction (CVF) on the mechanical properties of the constructs, we counted the cells and measured the CVF and the volume of the construct during time in culture. As a first approach we released live cells from the constructs by protease digestion. As shown in Fig. 5A, the cell number increased from 750,000 at the time of seeding the cultures to ~ 5 × 10^6^ at 2 days prior to 100% construction (i.e. T2). Therefore, the cells amplified ~ 6 fold during 4 days in fibrin. From T0, the number of cells decreased to a steady state value of ~ 1 × 10^6^ cells/construct. Further analysis showed that the decrease in cell numbers was the result of decreased cell-cycle rate (Fig. 5A). To determine the CVF we estimated the volume of cells and the volume of ECM from electron micrographs of the constructs. As shown in Fig. 5, the number of cells and the cross-sectional area of the constructs decreased between T0 and T7-to-T10, and then remained approximately constant until T42. The decrease in cellularity without loss of ECM presumably explained, in part, the consolidation in mechanical properties shown in Fig. 3. We noted that the collagen fibrils in T42 constructs were circular in cross-section and without signs of proteolytic degradation (see Fig. 4).

### 2.5 Viable cells are required for the development of strength and stiffness in tendon constructs

**Fig. 6:**
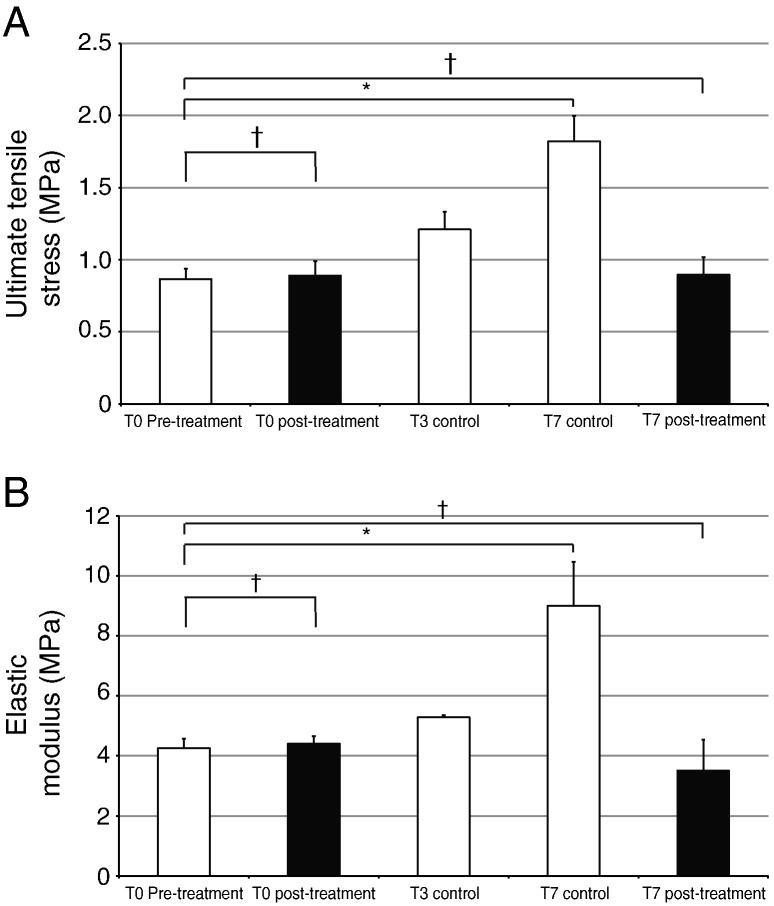
Triton X-100 treatment of T0 tendon constructs arrests development of mechanical properties. (A) Ultimate tensile stress (UTS) of the control tendon constructs increased 2-fold during T0 to T7. In contrast, the Triton-treated constructs showed no significant increase in UTS. (B) Elastic modulus of the control tendon constructs increased 2.2-fold during T0 to T7. The Triton-treated constructs, however, showed no significant change in the elastic modulus. ^⁎^*p* < 0.05, ^†^*p* > 0.2.

We wanted to know if the improvements in mechanical properties observed between T0 and T10 (see Fig. 3) were the result of cellular activity or changes in the ECM. Therefore tendon constructs were treated with Triton X-100 at T0 and left in culture for up to 7 days. Propridium iodide staining for light microscopy and, separately, electron microscopy analysis showed that treatment with Triton X-100 had removed the plasma membranes, as expected (data not shown). Tensile testing 1 h post-treatment and 7-days post-treatment with Triton demonstrated no change in mechanical strength or stiffness, in contrast to constructs that had not been treated with Triton X-100 (Fig. 6).

### 2.6 Cells in tendon constructs express non-muscle myosins

**Fig. 7:**
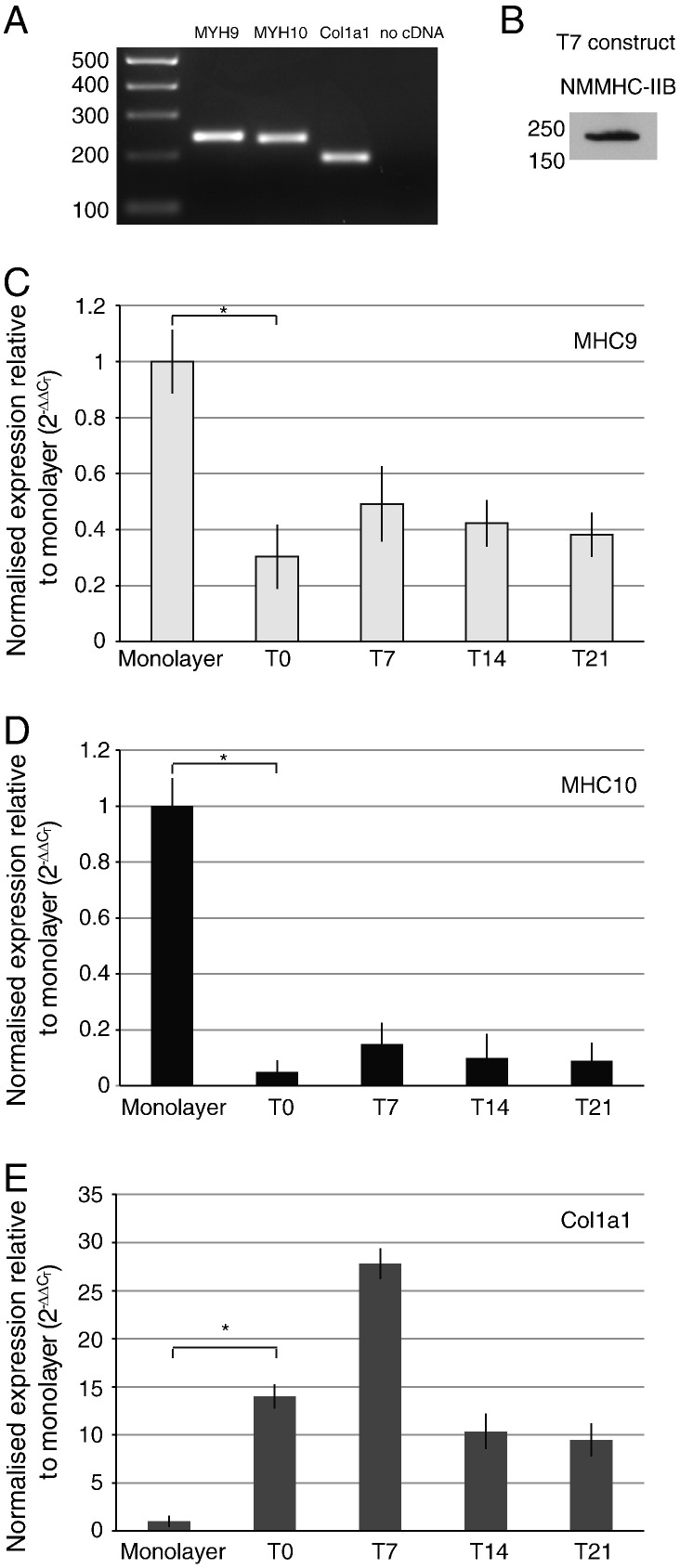
Expression of non-muscle MYH9 and MYH10 by cells in tendon constructs. (A) PCR gel electrophoresis analysis of MYH9 and MYH10 and *col1a1* gene expression. (B) Western blot for NMMHC-IIB protein. (C, D, E) Real-time PCR expression of MYH9 (C), MYH10 (D) and *col1a1* (E) in tendon constructs between T0 and T21. Expression of MYH9 and MYH10 was significantly reduced in tendon constructs compared to monolayer, whereas *col1a1* was significantly increased. ^⁎^*p* < 0.05.

PCR showed that the cells in the tendon constructs contained transcripts from MYH9 and MYH10, which encode the non-muscle myosin heavy chain proteins NMMHC-IIA and -IIB, respectively (Vicente-Manzanares et al., 2009) (Fig. 7A). An anti-NMMHC-IIA antibody was not available but western blot analysis showed the presence of NMMHC-IIB (Fig. 7B). Real-time PCR showed that the expression levels of MYH9 and 10 were highest in cells in monolayer than in constructs, presumably because the cells were under greater tension on tissue culture plastic than in constructs. However, transcript levels in constructs were maximal at T7 and were readily detectable for at least 21 days post-contraction. Analysis of *col1a1* (the gene encoding the α1(I) chain of type I collagen) showed that the levels of expression were significantly higher in cells in constructs (up to at least T21) than in cells cultured on tissue culture plastic.

### 2.7 An active actinomyosin complex is needed to generate the mechanical properties of tendon constructs

**Fig. 8:**
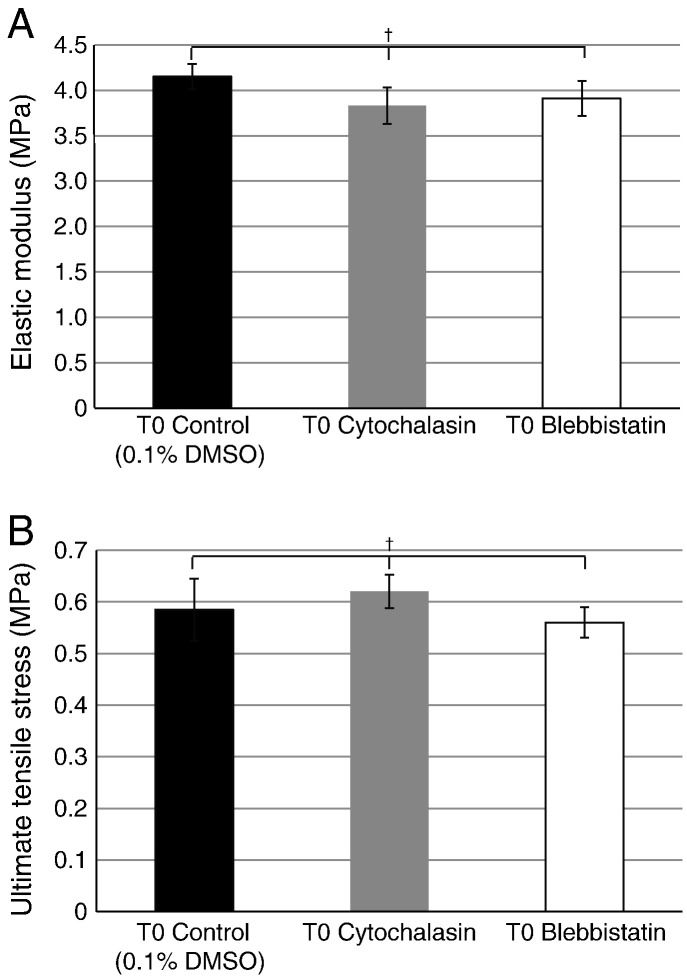
The effect of cytochalasin and blebbistatin treatment on the mechanical properties of tendon constructs at T0. Two-hour incubation with either cytochalasin or blebbistatin was immediately followed by mechanical testing. No significant differences in elastic modulus or UTS were seen between inhibitor treated constructs and control.

**Fig. 9:**
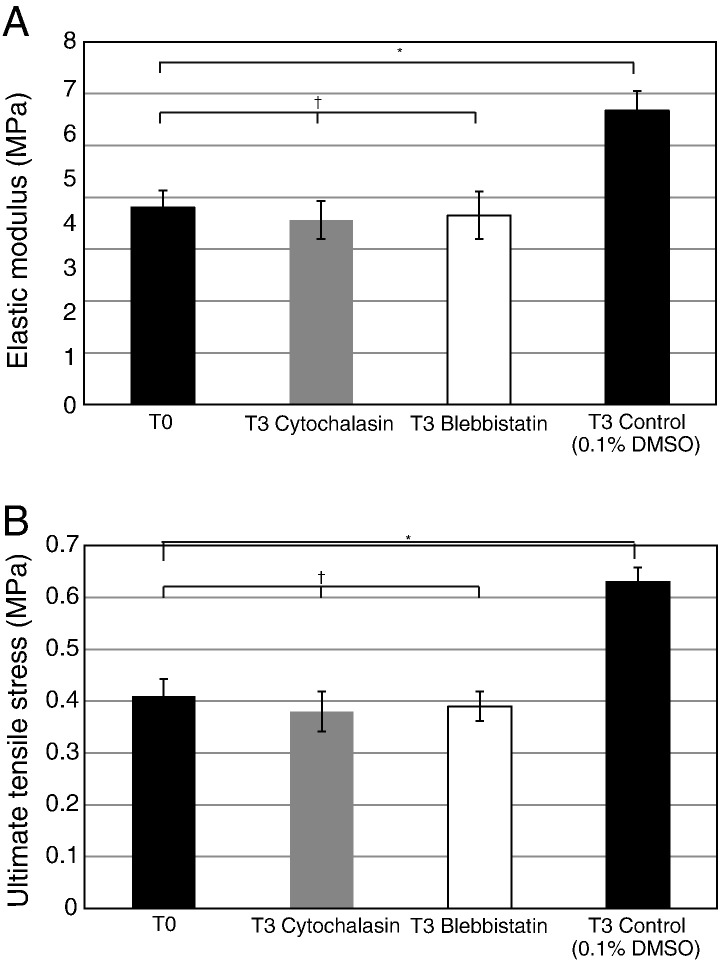
The effects of cytochalasin and blebbistatin on the development of mechanical properties of tendon constructs. T0 tendon constructs were incubated separately with cytochalasin and blebbistatin for 24 h and the constructs were incubated for a further 48 h in an inhibitor-free culture medium. Control samples contained 0.1% DMSO for the first 24 h. ^⁎^*p* < 0.05, ^†^*p* > 0.2.

Addition of cytochalasin or blebbistatin to the culture medium of 13-day ECMT cells in culture resulted in rounding up of the cells and persistent plasma membrane protrusions, respectively, as expected (data not shown). As shown in Fig. 8, addition of blebbistatin or cytochalasin to T0 constructs for 2 h had no significant effects on the UTS or elastic modulus of the constructs. Therefore, disruption of the actin cytoskeleton or the actinomyosin system had no effect on the mechanical properties of T0 constructs. However, a longer incubation with blebbistatin or cytochalasin (24 h) resulted in failed improvement of mechanical properties during a further 2 days in culture. As shown in Fig. 9, blebbistatin and cytochalasin inhibited the increases in UTS and elastic modulus during time in culture. Taken together, these results suggested that forces generated by the actinomyosin machinery contribute to the improvement of mechanical strength of the construct but not to the maintenance of mechanical properties.

### 2.8 Blebbistatin inhibits the cells' ability to contract the tendon construct

**Fig. 10:**
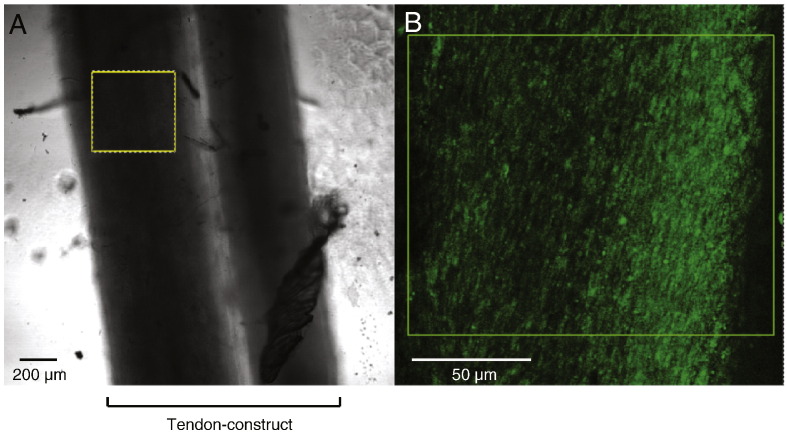
Demonstration of live cells post-treatment with blebbistatin. After 24-hour incubation with blebbistatin constructs were stained with calcein-AM. (A) A transmitted light image of a construct. (B) Confocal microscope image of the boxed area in (A). Live cells appear green.

**Fig. 11:**
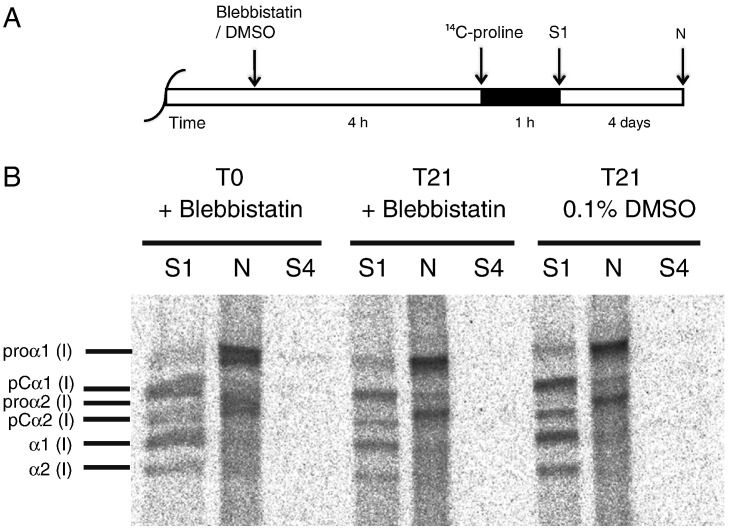
Continuous labeling with ^14^C-proline. (A) Tendon constructs (at T0 or at T21) were treated with either blebbistatin or 0.1% DMSO for 4 h and then ^14^C-proline was added for a further 1 h. Proteins were extracted as previously described (Canty et al., 2004). (B) Sequential salt extractions (S1, S4) for extracellular proteins followed by an NP-40 detergent extraction (N) for intracellular proteins demonstrated that blebbistatin did not affect the ability of the cells to secrete procollagen and to convert procollagen to collagen.

**Fig. 12:**
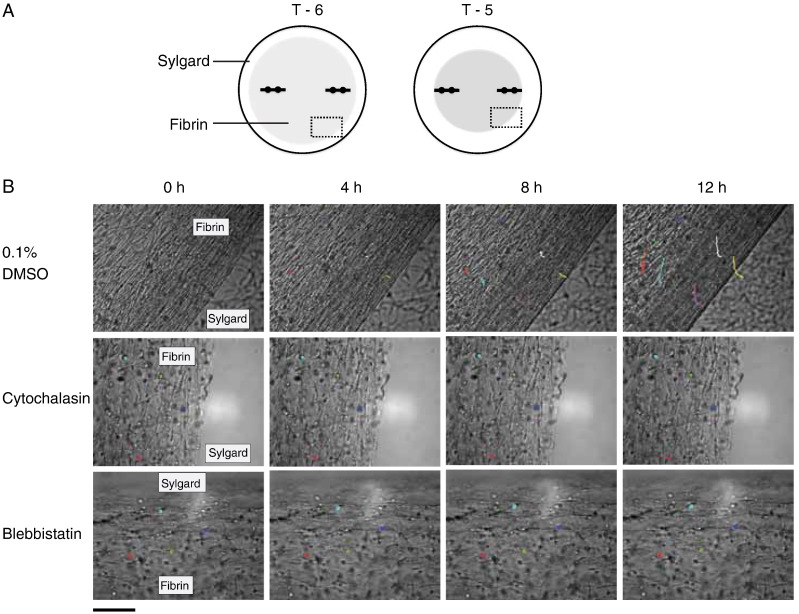
Live-cell imaging of cells in tendon constructs. Cells seeded in fibrin gels at T6 were treated with either DMSO, blebbistatin, or cytochalasin and imaged for 12 h at 5-min intervals. (A) Schematic showing the fibrin gel (shaded grey) contracting around the fixed-position pins. The box indicates the area imaged. (B) Cell contraction was highlighted with colored lines tracking the position of individual cells over time. Cell contraction was abolished by treatment with blebbistatin or with cytochalasin. Scale bar, 100 μm.

Examination of the constructs at T3 with calcein-AM stain demonstrated live cells in the constructs (Fig. 10). Furthermore, metabolic labeling with ^14^C-proline showed that blebbistatin did not noticeably affect the ability of the cells to secrete procollagen and to convert the procollagen to collagen (Fig. 11). Previous studies had shown that cytochalasin disrupts the actin cytoskeleton without significantly affecting the secretion of procollagen or the conversion of procollagen to collagen by 13-day ECMT cells (Canty et al., 2006). The presence of bands corresponding to pCα1(I) and α1(I) chains showed that procollagen was selectively processed by the procollagen N- and C-proteinases (Canty and Kadler, 2005). In the next series of experiments we used live-cell phase contrast microscopy to obtain new insights into how blebbistatin inhibited the improvement of mechanical properties in the tendon constructs. Fig. 12 shows still images from movies (see Supplementary Files) of the edges of constructs that are actively being contracted by cells. The data show that the edges of the construct move inwards as the construct changes shape from a 3D disc of fibrin to a cylindrical construct containing collagen fibrils in parallel register. Tracking of individual cells shows that the cells move in unison during contraction of the construct and do not change position relative to each other. Movies recorded from constructs treated with cytochalasin and blebbistatin show no contraction.

## 3 Discussion

The results presented here show that the tendon constructs assembled a fibrous matrix with mechanical properties similar to native embryonic tendons. Furthermore, constructs exhibited key aspects of tendon development: (i) a gradual decrease in cell/ECM volume ratio with time in culture to produce a CVF of ~ 0.3, (ii) the toe–linear–fail stress–strain curve typical of tendon, and (iii) collagen fibril diameters and FVF similar to those that occur in 13-day ECMT. Our ability to culture the tendon constructs with a viable cell population for up to 42 days permitted an initial study of the molecular mechanisms that generate the mechanical properties of the tendon-like matrix.

Previous studies have shown that the CVF in ECMT falls from 65% at day 10 to 47% at day 14 and to 36% at day 17, concomitant with a progressive increase in strength (ultimate tensile stress) and stiffness (modulus) (McBride et al., 1985, 1988). We showed here that the CVF of embryonic tendon constructs fell from 51% at T0 to 37% at T7 and 32% at T21 and stayed constant until T42. At the same time mechanical properties improved from T0 to T10 and stayed statistically the same until T42. As shown in Fig. 3, there was no significant drop in ultimate tensile strength and elastic modulus between T10 and T42. Furthermore, the mechanical properties at T42 were not significantly different from those of E13 chick metatarsal tendons. The mechanical measurement data collected at T21 appeared to stand out from the other measurements. However, the ultimate tensile strength and elastic modulus increased during T21 to T42. Furthermore, ^14^C-metabolic labeling and real-time PCR for *col1a1* showed that there was active procollagen synthesis in T21 constructs. Analysis of cell number in the constructs (by EM and cell counting) showed that the number of cells decreased during T2 to T14 and remained constant to T42 (as shown in Fig. 5). Reduced cell number was most likely the result of a decreased number of cells in S-phase. Taken together, our results and those of McBride and co-workers suggest that the increase in mechanical properties of embryonic tendon is the result of increased ECM/cell ratio (with additional contributions *in vivo* from progressive increase in fibril diameter and FVF as development proceeds) and the result of cell-derived forces exerted by the actinomyosin system on the ECM. It is likely that cyclical forces *in vivo* contribute to increased mechanical properties in native tendons. For example, Deng and co-workers report that static strain force provided by a U-shape spring over nine weeks increased collagen fibril diameter and mechanical strength of polyglycolic acid scaffolds seeded with adult fibroblasts (Deng et al., 2009).

An interesting observation was the increase in cell number during the initial formation of the tendon construct (i.e. from seeding the cells in fibrin (T-7) to T0) followed by a decrease in cell number as the ECM/cell ratio approximated that that occurs *in vivo*. FACS analysis showed that cells in fibrin actively divide (as shown by increased cell numbers and a higher proportion of cells in S-phase) at the time of active fibrinolysis and active collagen fibril assembly. Elastic modulus increased during T0 to T10 and then remained constant during the following 32 days in culture, and, was statistically indistinguishable from that recorded from 13-day chick embryonic tendons.

Constructs formed from adult fibroblasts (Calve et al., 2004) or bone marrow stromal cells (Hairfield-Stein et al., 2007) have similar mechanical and ultrastructural properties to the constructs prepared from 13-day ECMT cells (e.g. modulus of 15–17 MPa, UTS of ~ 2 MPa and relatively narrow-diameter collagen fibrils). Although adult cells can form embryonic-like tissue *in vitro*, the contraction process takes much longer than with embryonic tendon cells (three months versus 7 days), and with a lower success rate (~ 60% versus > 95%) (Calve et al., 2004). Furthermore, cells from adult patellar tendon cultured in the same 3D fibrin system used here produce fibrils of ~ 35 nm diameter (Bayer et al., 2010). It appears, therefore, that collagen fibril diameters are determined by the culture conditions and not by the species or developmental stage of the tendon cells.

This study used cells seeded in fibrin gels to ensure that ultrastructural and biomechanical measurements we made were of the *de novo* matrix assembled by the cells and not of matrix resulting from remodeled existing collagen. However, both free-floating and restrained collagen gels have previously been used to investigate the mechanisms of matrix contraction by fibroblasts (Abe et al., 2003; Grinnell, 2000). In floating gels in which cells are in a low cell-matrix tension state lysophosphatidic acid (LPA) stimulated contraction through a myosin II-independent pathway involving p21-activating kinase (PAK1) (Rhee and Grinnell, 2006; Rhee et al., 2007). However, LPA can also stimulate contraction by cells in a high cell-matrix tension state in restrained collagen gels through Rho kinase and myosin II (Rhee and Grinnell, 2007; Rhee et al., 2007). In the present study tendon constructs of fixed-length were used, and, therefore, it is possible that cell contraction is Rho kinase dependent in a similar mechanism to that seen in a restrained collagen gel. 3D collagen matrices have also been used to investigate collagen fibril flow (translocation) (Miron-Mendoza et al., 2008), demonstrating that cell forces can actively move and remodel extracellular collagen fibrils.

Studies using bioartificial tissue have also allowed investigation of the contribution of the cell to tissue mechanical properties (Marquez et al., 2010). Fibroblast populated collagen matrices were used because their mechanical behaviour closely resembles natural tissues (Marquez et al., 2010; Wakatsuki et al., 2000). Correlation between quantitative measurement of the force of cell contraction and changes in matrix stiffness has demonstrated the effect of cell force on matrix remodeling and mechanical properties (Wakatsuki and Elson, 2003). Wille et al. (2006) described the relative contribution of cell force and matrix force to tissue force generated by cardiac fibroblasts in collagen gels. Treatment with cytochalasin D disabled cellular force generation and showed that both the cell and the matrix contributed to gel contraction, and that their relative contribution changed with stimulation by cyclic stretch. Other studies using bioartificial tissue constructs have shown that tissue stiffness increases with cellular concentration and that this effect was due to increases in both cell- and matrix-contribution to gel stiffness (Marquez et al., 2006).

The demonstration that the mechanical properties of the tendon constructs are established during 7 days in culture and are then comparable to those of 13-day ECMT, allowed us to investigate the contribution of the actinomyosin system to the mechanical properties of the constructs. Real-time PCR showed the expression of MYH9 and 10, and western blot analysis showed the presence of NMMHC-IIB. Therefore, in subsequent experiments we used blebbistatin to inhibit the action of NMMII motors and cytochalasin to disassemble the actin cytoskeleton. We showed that these small compounds had no effect on the pre-established mechanical properties of the constructs *per se*. In contrast, treatment with blebbistatin and cytochalasin completely abolished the ability of the cells to *improve* the mechanical properties of the constructs with time in culture. Live-cell imaging with calcein-AM and ^14^C-proline metabolic labeling showed that the cells were viable in the presence of blebbistatin and that the cells synthesized procollagen and converted the procollagen to collagen. Therefore, the halt in improvement of mechanical properties in the presence of blebbistatin was not the result of decreased collagen synthesis or of decreased cell viability. Also, the active production of procollagen and the ultrastructure of the collagen fibrils by electron microscopy suggested that the constructs were not degrading or deteriorating with extended time in culture. In the next series of experiments we used live-cell phase contrast light microscopy to observe the cells during the formation of the constructs. The movies showed that the constructs form by contraction of the edge of the construct without overt cellular migration, and that the cells remained in the same relative position to each other. Presumably, the formation of the constructs and concomitant improvement in mechanical properties was the result of cells pulling on each other and/or on the ECM that surrounds them. In the presence of blebbistatin (or cytochalasin) the cells did not contract the gel. Close inspection of the movies showed that the cells were static. The inability of the cells to (i) contract the constructs and (ii) improve the mechanical properties of the constructs, in the presence of blebbistatin, shows that non-muscle myosin II-derived forces acting on the newly-synthesized matrix of narrow-diameter collagen fibrils are important for establishing the constructs and improving mechanical properties.

In conclusion, we have successfully generated a biomaterial (a bioartificial tendon construct) that has the biomechanical and ultrastructural features of embryonic tendon in that they comprise narrow-diameter collagen fibrils that are close packed, parallel to the long axis, and included within fibripositors that are seen in embryonic tendon. Analysis of cellular and mechanical properties suggests that 13-day ECMT cells have the potential to regulate the biomechanical properties of the ECM and the ECM/cell ratio *ex vivo*. The actinomyosin system is needed to generate the mechanical properties of the tendon construct but once established, the ECM maintains the mechanical properties in the absence of cellular activity. Tendon is required to be a taut tissue, with highly organized parallel arrays of collagen fibrils, for it to function mechanically. Presumably NMMII-mediated forces are required either to align the collagen fibrils, stabilize the alignment, or continually monitor the material properties of the pericellular matrix in response to inside-out and outside-in mechanical stimuli. Finally, the tendon constructs formed with fibrin as a provisional matrix provide a new biomaterial for studies of how embryonic tendon cells synthesize an ECM *de novo*, as well as a starting point from which to explore the molecular and physical mechanisms that generate the mechanical properties of tendon.

## 4 Materials and methods

### 4.1 Cell isolation and tendon-construct formation

Tendon constructs were assembled as previously described (Kapacee et al., 2008). Briefly, day-13 ECMT cells were propagated (not exceeding passage 7) in monolayer in DMEM4 culture medium (Sigma) supplemented with penicillin (100 U/mL), streptomycin (100 μg/mL; Lonza), l-glutamine (2 mM; Lonza), and fetal calf serum (10%; Sigma) until sufficient numbers of cells were available to form constructs. Cells were removed from tissue culture flasks using trypsin–EDTA (Lonza). Each well of a six well plate was lined with 2 mL of Sylgard (type 184 silicone elastomer, Dow Chemical, Midland, MI, USA) and incubated at 55 °C for 15 h to set. Two 0.1 mm minutien pins (Austerlitz, Czech Republic) were each put through one 0.25 cm length of suture (Ethicon) and inserted with a 1 cm gap in the Sylgard. Plates were sterilised by immersion for 1 h in 100% ethanol under UV light. 6.15 × 10^5^ cells were suspended in 400 μL of complete medium plus 83 μL of 20 mg/mL fibrinogen and 10 μL of 200 U/mL thrombin (both bovine; Sigma, St. Louis, MO, USA), deposited in each well and incubated at 37 °C, 5% CO_2_. After a 5-min setting time, cell-matrix layers were ‘scored’ with a fine pipette tip to prevent adhesion to the side of the well, then incubated with 5 mL culture medium (as above) supplemented with l-ascorbic acid 2-phosphate (200 μM; Lonza). The gel was scored every two days until, at approximately seven days post seeding, it had contracted to form a linear construct between the pinned sutures. The timepoint of ‘contraction’ was defined as T0.

### 4.2 Mechanical testing

Constructs were removed from culture at contraction (T0) and 3, 7, 10, 14, 21, 28, 35 and 42 days post-contraction (T3, T7, T10, T12, T21, T28, T35 and T42, respectively). A minimum of ten tendon constructs were tested per time point. Because it was not possible to take histological sections of constructs before mechanical testing (which would damage the tissue) diameters were measured from digital photographs. Construct diameter was then used to calculate transverse area according to the formula πd. This assumed a circular transverse shape, as demonstrated previously from histological sections (Kapacee et al., 2008) and used in mechanical testing of tissue engineered ligament (Hairfield-Stein et al., 2007). An average of three diameter measurements was recorded. Tendon constructs were mounted on a supportive frame of coarse (grade 100) sandpaper using super glue (as performed by McBride et al. (1988) (see Fig. 1)). The mounting frame was clamped in an Instron 4301 mechanical testing machine fitted with a 100 N load cell (Instron Inc., High Wycombe, UK). The clamps were hand-tightened, and samples were excluded from analysis if they slipped during testing. After clamping, the side-pieces of the frame were cut to prevent stretching or damage of the tendon construct. The original contour length (L_O_) of tendon constructs was measured from a digital photograph of the mounted construct (see Fig. 1). A tare load of 10 mN was applied at the start of the tensile test to fully straighten the tendon construct. The length at failure was determined from the Instron test (giving change in length L_∆_). Tendon constructs were tested to failure with a strain rate of 5 mm per minute (equivalent to approximately 1% strain per second). Tensile testing of day-13 ECMT, cut into 1.5 cm lengths, was also performed using this method.

### 4.3 Analysis of output from mechanical tests

Instron series XI software produced a force extension curve from which it was possible to calculate ultimate tensile stress (UTS; MPa; calculated from: maximum force (N) / transverse area (m^2^)), and failure strain (change in length at failure/original length). Elastic modulus (E; MPa) was calculated as the gradient of the linear portion of the stress–strain curve.

### 4.4 Electron microscopy

#### 4.4.1 Tissue fixation and embedding

For ultrastructural analysis a minimum of 2 constructs were examined for each time point (4 samples at T0 and T7 and 3 at T14). Constructs were immersed in primary fixative (100 mM sodium phosphate buffer (pH 7.0) containing 2% glutaraldehyde (Agar Scientific)) for 30 min at room temperature, then cut up into three smaller pieces and placed in fresh primary fixative for 2 h at 4 °C. Samples were transferred to secondary fixative (50 mM sodium phosphate buffer (pH 6.2) containing 2% glutaraldehyde and 1% osmium tetroxide (Agar Scientific)) for 40 min at 4 °C before being thoroughly washed with distilled water and *en bloc* stained in 1% aqueous uranyl acetate for 16 h at 4 °C. Constructs were dehydrated in graded acetone (30%, 50%, 70%, and 90%) followed by four changes of 100% acetone for 10 min each at room temperature. Samples were treated with propylene oxide for 10 min at room temperature then infiltrated with a mixture of TAAB low viscosity resin (medium hardness; Agar Scientific) and propylene oxide. Samples were put into 50% resin on a rotator overnight at room temperature. The sample was then incubated in 70%, 90% and three changes of 100% resin, each for 1 h at room temperature. Samples were put in resin moulds for polymerization at 60 °C for 24 h.

#### 4.4.2 Sectioning and staining

Ultra-thin sections were taken on a Reichert-Jung Ultracut (Leica Microsystems, UK) ultramicrotome using a diamond knife (Druker International, NL). Sections were stained with 2% uranyl acetate in 70% ethanol for 20 min then washed in distilled water. Sections were counter stained in 0.3% lead citrate in 0.1 M NaOH for 5 min and washed in distilled water.

#### 4.4.3 Micrograph capture, sampling technique and ultrastructural measurements

Sections were examined using an FEI Tecnai 12 Twin Transmission Electron Microscope (TEM). Images were captured using a 2 k × 2 k cooled CCD camera (F214A, Tietz Video and Image Processing Systems, Gauting, Germany). For each sample a minimum of three different sections were reviewed. A thorough sampling of each section was performed. Three different magnifications were used: 2100× for cell volume fraction (giving a large-area survey), 6800× for fibril volume fraction and 11,000× for fibril diameter. The sampling procedure generated 20 images of each section at 2100×, 40 views per section at 6800× and 60 views per section at 11,000×. This sampling method enabled representative EM quantification of the collagenous matrix.

Magnification calibration was performed for each magnification using a diffraction-grating replica grid (2160 lines/mm, Agar Scientific, Stansted, UK). All measurements were made using ImageJ software (NIH freeware, http://rsb.info.nih.gov/nih-image). Cell volume fraction was calculated from the proportion of the transverse area occupied by cells (total transverse area / cell area). Fibril volume fraction was calculated from the proportion of transverse area occupied by collagenous fibrils (total transverse area / collagen fibril area).

### 4.5 Cell counts and cell-cycle analysis

Three construct samples per timepoint were used for cell counts and 3 samples for cell-cycle analysis. Cell culture medium was removed from tendon constructs and washed twice in PBS. Tendon constructs were digested in 10 mL 0.25% trypsin (Invitrogen) plus 80 mg bacterial collagenase type IV (210 U/mL; Worthington, USA). Digestion was for 1.5 h at 37 °C. Live-cell counts were performed on 10 μL of cell suspension in 40 μL trypan blue solution (Sigma) using a hemocytometer.

For cell-cycle analysis, cells were collected by centrifugation (13,000 × *g*, 5 min), washed in PBS, and resuspended in 500 μL PBS. The cell suspension was mixed with 5 mL 70% −20 °C ethanol and stored at 4 °C, if necessary. Prior to fluorescence activated cell sorting (FACS) analysis the cells were collected by centrifugation (13000 × *g*, 5 min), washed in PBS and then resuspended in 300 μL propridium iodide solution (38 mM sodium citrate pH 7.4; Sigma), treated with 10 μL RNAse solution (20 mg/mL; Invitrogen) and incubated at 37 °C for 30 min. FACS was performed using a CyAn ADP flow cytometry analyser (Beckman Coulter, USA) and processed using Modfit LT software (Verity Software House, USA).

### 4.6 Treatment with Triton solution, cytochalasin and blebbistatin

Ten constructs per group were used at each timepoint. Triton solution was prepared from 0.5% Triton X-100 solution (Sigma) in 40 mL PBS with 2 protease inhibitor tablets (Sigma). Constructs were washed with PBS twice, incubated with Triton solution for 20 min (2 mL per construct), washed with PBS, and incubated with fresh Triton solution for 20 min. Constructs were washed with PBS, and incubated in the culture medium supplemented with FCS (as above) at 37 °C and 5% CO_2_. In control samples, constructs were treated similarly but without the addition of Triton to the PBS.

Two experiments were performed with inhibitors of the actinomyosin complex. Newly formed constructs (T0) were incubated for either 2 h or 24 h with either blebbistatin (25 μM, 0.1% v/v dimethylsulfoxide (DMSO); Pfizer, UK) or cytochalasin (10 μM, 0.1% v/v DMSO; Calbiochem, NJ, USA) at 37 °C in 5% CO_2_. Two-hour incubation samples were then taken for mechanical testing immediately, and 24-hour samples examined two days later at T3. Control samples were incubated in 0.1% v/v DMSO. Constructs were also examined post-incubation with Calcein-AM (10 μM for 2 h; Sigma) to detect live cells by light microscopy. Images were collected on a Leica TCS SP2 AOBS inverted confocal microscope using a 20× HCX PL Fluotar objective. The confocal settings were pinhole 1 airy unit, scan speed 1000 Hz unidirectional, format 1024 × 1024. Images were collected using the following detection mirror settings: excitation 496 nm (20%) and emission 500–540 nm.

### 4.7 Molecular biology

#### 4.7.1 Primer sequences

The following primers were used: chicken MYH9 forward primer, ACGCGTACCTCCAGAGAAGA; reverse primer, GGATAGCACAGCTGGAGGAG; chicken MYH10 forward primer, CTGAGGACAAAACGTGAGCA; reverse primer, GAAGTGAAGGTGTTGCAGCA; chicken Col1a1 forward primer, CAGCCGCTTCACCTACAGC; reverse primer, TTTTGTATTCAATCACTGTCTTGCC (Martin et al., 2001); chicken ribosomal 18s forward primer, GTAACCCGTTGAACCCCATT; reverse primer, CTACTACCGATTGGATGG.

#### 4.7.2 RNA isolation from cultured chick tenocytes

Total RNA was extracted from subconfluent (~ 80%) cultures of fibroblasts using TRIzol reagent followed by DNase treatment.

#### 4.7.3 RNA isolation from tendon constructs and RT-PCR

Tendon constructs were rinsed briefly in PBS. TRIzol reagent (Invitrogen, Carlsbad, CA) was added and the tissue rapidly frozen in liquid nitrogen prior to disruption using a Mikro-Dismembrator (Sartorius) (twice at 2000 rpm for 90 s). RNAs were extracted from the tissue following the manufacturer's instructions (Invitrogen, Carlsbad, CA) followed by DNase treatment. cDNA was transcribed from 2 μg of RNA with TaqMan reverse transcriptase (RT) polymerase (Applied Biosystems), using an oligo(dT)16 primer. RT-PCR analysis was performed on mRNAs by using 20-mer primers complementary to MYH9, MYH10, col1a1 and ribosomal 18s from the chick. Amplification of the correctly sized products was verified by electrophoresis on a 2% Tris–borate–EDTA gel. The identities of the product were confirmed by DNA sequencing.

#### 4.7.4 Sequencing of RT-PCR fragments

PCR products (5 ng) were sequenced using a BigDye Terminator v3.1 cycle sequencing kit (Applied Biosystems). Samples were placed in a thermal cycler under the following conditions: initial denaturation was performed at 96 °C for 1 min, followed by 25 cycles of 96 °C for 10 s, 50 °C for 5 s, and 60 °C for 4 min. Samples were precipitated with ethanol–sodium acetate prior to analysis.

#### 4.7.5 Quantitative PCR

RNA was extracted and cDNA synthesized as described above. A total of 50 ng cDNA was loaded per qPCR well. Quantitative RT-PCR was performed using Chromo4 (BioRad) with SYBR Green (Eurogentec). Output was analysed using Opticon monitor 3 software (BioRad). Results were normalized to ribosomal 18s and four samples were used for each time point (Schmittgen and Livak, 2008).

### 4.8 Western blot analyses

Total protein was extracted from tendon constructs by incubation in RIPA buffer overnight on a shaker at 4 °C. Protein extracts were examined by standard western blot procedures and optimal antibody dilutions determined empirically. Anti-MHY10 antibodies (clone CMII 23) were obtained from Developmental Studies, Hybridoma Bank, University of Iowa.

### 4.9 Collagen ^14^C-proline labeling

Continuous labeling experiments were performed at 37 °C in DMEM4 containing 1% (vol/vol) PS, 2 mM l-glutamine, 200 μM ascorbate, and 400 μM βAPN, and supplemented with 2.5 μCi/mL of ^14^C-proline and 25 μM blebbistatin as required. Labeling was stopped by transferring the tendons to 25 mM EDTA, and 50 mM Tris–HCl, pH 7.5, at 4 °C. Tendon constructs subjected to continuous labeling analysis in 1 mL aliquots of the supplemented medium were extracted in 100 μL aliquots of salt extraction buffer (1 M NaCl, 25 mM EDTA, and 50 mM Tris–HCl, pH 7.4) containing protease inhibitors and supplemented as required with 1% NP-40 detergent as previously described (Canty et al., 2004, 2006). Tendon constructs were extracted in four changes of salt extraction buffer: overnight (S1), 6 h (S2), overnight (S3), 6 h (S4), and an overnight in NaCl extraction buffer containing NP-40 (N). Extracts were analysed on 4% precast SDS polyacrylamide gels (Invitrogen) under reducing conditions. The gels were fixed in 10% methanol and 10% acetic acid, dried under vacuum, and exposed to a phosphorimaging plate (Fuji BAS-III or BAS-MS). After overnight exposure the phosphorimaging plates were processed using a phosphorimager (Fuji BAS 2000 or 1800).

### 4.10 Live-cell imaging of constructs

Tendon constructs at T6 were treated with either 0.1% DMSO, 10 μM cytochalasin or 25 μM blebbistatin and imaged using an AS MDW live-cell imaging system (Leica) with a 20×/0.5 HC Plan Fluotar objective. Point visiting was used to allow multiple positions to be imaged within the same time-course and cells were maintained at 37 °C and 5% CO_2_. Images were collected every 5 min for 12 h using a Cascade II EM CCD camera for ultra sensitive imaging (Photometrics). Cell tracking was performed using the Particle Analysis manual tracking plugin for ImageJ.

### 4.11 Statistical analysis

Statistical analysis was performed using SPSS version 14. Mechanical data (UTS, modulus and failure strain) and ultrastructural data (fibril diameter, cell volume fraction and fibril volume fraction) were examined using 1-way ANOVA. Quantitative PCR data was examined using the Wilcoxon two group test. Significance was set at the *p* < 0.05 level. Data are presented as mean ± standard error of the mean unless otherwise stated.

The following are the supplementary materials related to this article.

Supplementary File 1

Supplementary File 2

Supplementary File 3

## References

[bb0005] Abe M., Ho C.H., Kamm K.E., Grinnell F. (2003). Different molecular motors mediate platelet-derived growth factor and lysophosphatidic acid-stimulated floating collagen matrix contraction. J. Biol. Chem..

[bb0010] Allingham J.S., Smith R., Rayment I. (2005). The structural basis of blebbistatin inhibition and specificity for myosin II. Nat. Struct. Mol. Biol..

[bb0015] Askari J.A., Buckley P.A., Mould A.P., Humphries M.J. (2009). Linking integrin conformation to function. J. Cell Sci..

[bb0020] Bank R.A., TeKoppele J.M., Oostingh G., Hazleman B.L., Riley G.P. (1999). Lysylhydroxylation and non-reducible crosslinking of human supraspinatus tendon collagen: changes with age and in chronic rotator cuff tendinitis. Ann. Rheum. Dis..

[bb0025] Bayer M.L., Yeung C.Y., Kadler K.E., Qvortrup K., Baar K., Svensson R.B., Magnusson S.P., Krogsgaard M., Koch M., Kjaer M. (2010). The initiation of embryonic-like collagen fibrillogenesis by adult human tendon fibroblasts when cultured under tension. Biomaterials.

[bb0030] Birk D.E., Trelstad R.L. (1985). Fibroblasts create compartments in the extracellular space where collagen polymerizes into fibrils and fibrils associate into bundles. Ann. NY Acad. Sci..

[bb0035] Birk D.E., Trelstad R.L. (1986). Extracellular compartments in tendon morphogenesis: collagen fibril, bundle, and macroaggregate formation. J. Cell Biol..

[bb0040] Calve S., Dennis R.G., Kosnik P.E., Baar K., Grosh K., Arruda E.M. (2004). Engineering of functional tendon. Tissue Eng..

[bb0045] Canty E.G., Kadler K.E. (2005). Procollagen trafficking, processing and fibrillogenesis. J. Cell Sci..

[bb0050] Canty E.G., Lu Y., Meadows R.S., Shaw M.K., Holmes D.F., Kadler K.E. (2004). Coalignment of plasma membrane channels and protrusions (fibripositors) specifies the parallelism of tendon. J. Cell Biol..

[bb0055] Canty E.G., Starborg T., Lu Y., Humphries S.M., Holmes D.F., Meadows R.S., Huffman A., O'Toole E.T., Kadler K.E. (2006). Actin filaments are required for fibripositor-mediated collagen fibril alignment in tendon. J. Biol. Chem..

[bb0060] Deng D., Liu W., Xu F., Yang Y., Zhou G., Zhang W.J., Cui L., Cao Y. (2009). Engineering human neo-tendon tissue *in vitro* with human dermal fibroblasts under static mechanical strain. Biomaterials.

[bb0065] Egerbacher M., Arnoczky S.P., Caballero O., Lavagnino M., Gardner K.L. (2008). Loss of homeostatic tension induces apoptosis in tendon cells: an *in vitro* study. Clin. Orthop. Relat. Res..

[bb0070] Fessel G., Snedeker J.G. (2009). Evidence against proteoglycan mediated collagen fibril load transmission and dynamic viscoelasticity in tendon. Matrix Biol..

[bb0075] Grinnell F. (2000). Fibroblast-collagen-matrix contraction: growth-factor signalling and mechanical loading. Trends Cell Biol..

[bb0080] Grinnell F., Petroll W.M. (2010). Cell motility and mechanics in three-dimensional collagen matrices. Annu. Rev. Cell Dev. Biol..

[bb0085] Hairfield-Stein M., England C., Paek H.J., Gilbraith K.B., Dennis R., Boland E., Kosnik P. (2007). Development of self-assembled, tissue-engineered ligament from bone marrow stromal cells. Tissue Eng..

[bb0090] Harris A.K., Stopak D., Warner P. (1984). Generation of spatially periodic patterns by a mechanical instability: a mechanical alternative to the Turing model. J. Embryol. Exp. Morphol..

[bb0095] Huang Y.C., Dennis R.G., Larkin L., Baar K. (2005). Rapid formation of functional muscle *in vitro* using fibrin gels. J. Appl. Physiol..

[bb0100] Huang Y.C., Dennis R.G., Baar K. (2006). Cultured slow vs. fast skeletal muscle cells differ in physiology and responsiveness to stimulation. Am. J. Physiol. Cell Physiol..

[bb0105] Huang Y.C., Khait L., Birla R.K. (2007). Contractile three-dimensional bioengineered heart muscle for myocardial regeneration. J. Biomed. Mater. Res. A.

[bb0110] Humphries J.D., Byron A., Humphries M.J. (2006). Integrin ligands at a glance. J. Cell Sci..

[bb0115] Jay P.Y., Pham P.A., Wong S.A., Elson E.L. (1995). A mechanical function of myosin II in cell motility. J. Cell Sci..

[bb0120] Kapacee Z., Richardson S.H., Lu Y., Starborg T., Holmes D.F., Baar K., Kadler K.E. (2008). Tension is required for fibripositor formation. Matrix Biol..

[bb0125] Lavagnino M., Arnoczky S.P. (2005). *In vitro* alterations in cytoskeletal tensional homeostasis control gene expression in tendon cells. J. Orthop. Res..

[bb0130] Limouze J., Straight A.F., Mitchison T., Sellers J.R. (2004). Specificity of blebbistatin, an inhibitor of myosin II. J. Muscle Res. Cell Motil..

[bb0135] Marquez J.P., Genin G.M., Pryse K.M., Elson E.L. (2006). Cellular and matrix contributions to tissue construct stiffness increase with cellular concentration. Ann. Biomed. Eng..

[bb0140] Marquez J.P., Elson E.L., Genin G.M. (2010). Whole cell mechanics of contractile fibroblasts: relations between effective cellular and extracellular matrix moduli. Philos. Transact. A Math. Phys. Eng. Sci..

[bb0145] Martin I., Jakob M., Schafer D., Dick W., Spagnoli G., Heberer M. (2001). Quantitative analysis of gene expression in human articular cartilage from normal and osteoarthritic joints. Osteoarthritis Cartilage.

[bb0150] McBride D.J., Hahn R.A., Silver F.H. (1985). Morphological characterization of tendon development during chick embryogenesis: measurement of birefrigence retardation. Int. J. Biol. Macromol..

[bb0155] McBride D.J., Trelstad R.L., Silver F. (1988). Structural and mechanical assessment of developing chick tendon. Int. J. Biol. Macromol..

[bb0160] Miron-Mendoza M., Seemann J., Grinnell F. (2008). Collagen fibril flow and tissue translocation coupled to fibroblast migration in 3D collagen matrices. Mol. Biol. Cell.

[bb0165] Ng G.Y., Oakes B.W., Deacon O.W., McLean I.D., Eyre D.R. (1996). Long-term study of the biochemistry and biomechanics of anterior cruciate ligament-patellar tendon autografts in goats. J. Orthop. Res..

[bb0170] O'Brien T.D., Reeves N.D., Baltzopoulos V., Jones D.A., Maganaris C.N. (2010). Mechanical properties of the patellar tendon in adults and children. J. Biomech..

[bb0175] Parry D.A., Barnes G.R., Craig A.S. (1978). A comparison of the size distribution of collagen fibrils in connective tissues as a function of age and a possible relation between fibril size distribution and mechanical properties. Proc. R. Soc. Lond. B Biol. Sci..

[bb0180] Rhee S., Grinnell F. (2006). P21-activated kinase 1: convergence point in PDGF- and LPA-stimulated collagen matrix contraction by human fibroblasts. J. Cell Biol..

[bb0185] Rhee S., Grinnell F. (2007). Fibroblast mechanics in 3D collagen matrices. Adv. Drug Deliv. Rev..

[bb0190] Rhee S., Jiang H., Ho C.H., Grinnell F. (2007). Microtubule function in fibroblast spreading is modulated according to the tension state of cell-matrix interactions. Proc. Natl Acad. Sci. USA.

[bb0195] Richardson S.H., Starborg T., Lu Y., Humphries S.M., Meadows R.S., Kadler K.E. (2007). Tendon development requires regulation of cell condensation and cell shape via cadherin-11-mediated cell–cell junctions. Mol. Cell. Biol..

[bb0200] Robinson P.S., Lin T.W., Reynolds P.R., Derwin K.A., Iozzo R.V., Soslowsky L.J. (2004). Strain-rate sensitive mechanical properties of tendon fascicles from mice with genetically engineered alterations in collagen and decorin. J. Biomech. Eng..

[bb0205] Schmittgen T.D., Livak K.J. (2008). Analyzing real-time PCR data by the comparative C(T) method. Nat. Protoc..

[bb0210] Starborg T., Lu Y., Huffman A., Holmes D.F., Kadler K.E. (2009). Electron microscope 3D reconstruction of branched collagen fibrils *in vivo*. Scand. J. Med. Sci. Sports.

[bb0215] Steinberg B.M., Smith K., Colozzo M., Pollack R. (1980). Establishment and transformation diminish the ability of fibroblasts to contract a native collagen gel. J. Cell Biol..

[bb0220] Tuan T.L., Grinnell F. (1989). Fibronectin and fibrinolysis are not required for fibrin gel contraction by human skin fibroblasts. J. Cell. Physiol..

[bb0225] Vicente-Manzanares M., Ma X., Adelstein R.S., Horwitz A.R. (2009). Non-muscle myosin II takes centre stage in cell adhesion and migration. Nat. Rev. Mol. Cell Biol..

[bb0230] Wakatsuki T., Elson E.L. (2003). Reciprocal interactions between cells and extracellular matrix during remodeling of tissue constructs. Biophys. Chem..

[bb0235] Wakatsuki T., Kolodney M.S., Zahalak G.I., Elson E.L. (2000). Cell mechanics studied by a reconstituted model tissue. Biophys. J..

[bb0240] Wille J.J., Elson E.L., Okamoto R.J. (2006). Cellular and matrix mechanics of bioartificial tissues during continuous cyclic stretch. Ann. Biomed. Eng..

